# Cumulus cells protect the oocyte against saturated free fatty acids

**DOI:** 10.21451/1984-3143-AR2018-0063

**Published:** 2018-08-03

**Authors:** Hilde Aardema, Peter L.A.M. Vos, Bart M. Gadella

**Affiliations:** Department of Farm Animal Health, Faculty of Veterinary Medicine, Utrecht University, The Netherlands

**Keywords:** cumulus, fatty acid, oocyte.

## Abstract

In the cow a major characteristic of metabolic stress is an elevated level of plasma free fatty acid, due to increased lipid mobilization from adipose tissue. Elevated levels of free fatty acids in blood (complexed to albumin) are associated with increased lipotoxicity in non-adipose tissue. An overview is provided on the negative impact of free fatty acids and the metabolic stress imposed on the oocyte and early embryo and thus on bovine fertility. There is increasing evidence that *in vitro* as well as *in vivo* the elevated levels of free fatty acids in blood during metabolic stress can severely hamper oocyte and embryo development. However, fatty acids do also form an essential nutrient source for the oocyte and embryo, which indicates that these good and bad effects of fatty acids should be in subtle balance to optimize the developmental competence of the oocyte and embryo.

## Introduction

This manuscript presents an overview of the current knowledge of fatty acid transfer from blood to the follicle, from the follicular fluid towards the cumulus-oocyte-complex and distribution and use of fatty acids in the oocyte. Cumulus cells appear to play an important role in both fatty acid transfer towards the oocyte and protection of the oocyte against elevated levels of free fatty acids during metabolic stress conditions (Aardema *et al*., 2013; Lolicato *et al*., 2015; Del Collado *et al*., 2017). In particular, saturated free fatty acids appear to have a detrimental impact on oocyte developmental competence (Leroy *et al*., 2005; Wu *et al*., 2010; Aardema *et al*., 2011).

Previously, it has been shown that the negative impact of saturated free fatty acids on oocytes can be compensated by mono-unsaturated oleic acid, which is present in the follicular fluid at high concentration (Aardema *et al*., 2013, 2015). Interestingly, cumulus cells appear to protect the oocyte by converting the potentially toxic saturated fatty acid into mono- unsaturated fatty acid, which is due to Stearoyl-CoA desaturase (SCD) activity and is followed by a safe storage of fatty acids by esterifying them into triacylglycerides (TAG) in lipid droplets (Aardema *et al*., 2017).

Fatty acids are an important nutrient source and building block for the developing oocyte and embryo (Sturmey *et al*., 2009; McKeegan and Sturmey, 2012). A tight fatty acid regulation via the surrounding cumulus cells appears to be a prerequisite for good oocyte developmental competence. The protective properties of the cumulus cells surrounding the oocyte appear to be particularly important during metabolic stress conditions when elevated levels of free fatty acids occur in the follicular fluid.

## Fatty acids in metabolic disorders

Metabolic disorders pose one of the major recent health concerns in society, with a high percentage and still growing number of obese and diabetes II patients. Metabolic stress conditions are associated with various medical implications, including impaired fertility in both human and animal. Currently, in the USA nearly half of the women in the fertile age are overweight, with an obesity incidence of more than 20% (Vahratian, 2009; Broughton and Moley, 2017). Since elevated levels of free fatty acids (FFA) are a major characteristic of metabolic stress conditions, which are due to elevated mobilization of body fat reserves, there has been a growing attention for the impact of FFA on oocyte quality and hence early embryonic development, both *in vivo* and *in vitro*. Therefore, the involvement of fatty acids in affecting the developmental competence of the bovine oocyte and embryo is the central topic of this review.

## The fatty acid environment of the oocyte

**Figure 1:**
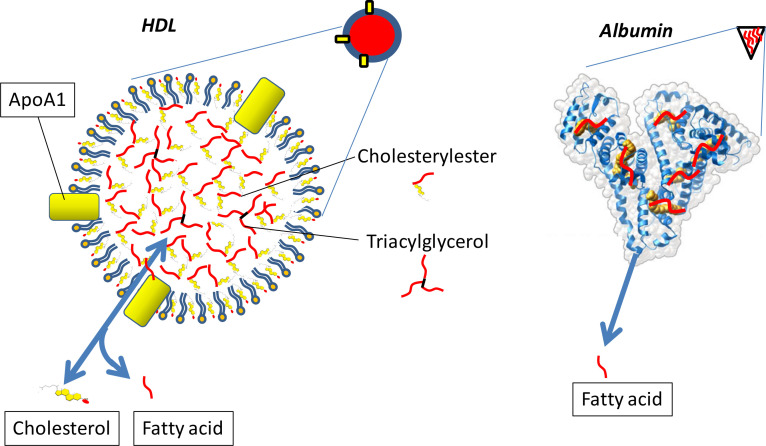
Lipid structure of HDL and the fatty acid albumin complex. HDL is assembled in the liver and secreted in blood cholesterol and fatty acids are exchanged on the surface of cells. Albumin is synthesized in the liver and is secreted in blood, it obtains fatty acids that originate from TAG from adipose tissues and are released after lipolysis and complexed to circulatory albumin. The fatty acid albumin complex can deliver these fatty acids to target cells. Albumin and HDL can both pass the blood-follicle membrane, which likely occurs via transcytosis. The shorthand depictions (diagonal to the right) are used in Fig. 2 to explain lipid delivery from these lipoprotein complexes to the cumulus oocyte complex.

Already from the secondary follicular growth stage onwards, oocytes contain neutral lipids, TAG and cholesteryl-esters, stored in lipid droplets. The number of lipid droplets present in the ooplasm of the oocyte progressively increases during oocyte growth (Fair *et al*., 1997). The origin of the stored neutral lipid in the oocyte is not completely known: neutral lipids may be exogenously derived after uptake from the environment as the receptor CD36 for fatty acid uptake is present in both cumulus cells and oocytes, but moreover oocytes contain the metabolic machinery for fatty acid synthesis, thus lipids may also be of endogenous origin (Cetica *et al*., 2002; Auclair *et al*., 2013; Uzbekova *et al*., 2015). Potential exogenous sources of fatty acids for the oocyte are present in the form of lipoproteins (mainly high- density lipoprotein; HDL), and FFA (fatty acids complexed to albumin) in follicular fluid (Fig. 1). FFA are mobilized from adipose tissue and transfer fatty acids to cells. Follicular fluid reflects the FFA levels of blood in human and bovine, but the free fatty acid compositions differ (Leroy *et al*., 2005; Jungheim *et al*., 2011; Yang *et al*., 2012; Aardema *et al*., 2013, 2015; Valckx *et al*., 2014). Metabolites in follicular fluid that originate from blood need to pass the blood-follicle barrier, formed by theca cells, the basal membrane and granulosa cells; a successful passage depends on both size and charge of the metabolite (Gosden *et al*., 1988; Fortune, 1994; Jaspard *et al*., 1997). Plasma lipoproteins are not able to pass the blood-follicle barrier, except for the high-density lipoprotein (HDL), the smallest lipoprotein subclass in particle diameter. Nevertheless, follicular fluid contains low amounts of very low- density lipoprotein (VLDL), which are significantly larger than HDL. Interestingly, VLDL amounts do not correlate with the levels in blood and appear to be secreted by granulosa cells (Gautier *et al*., 2010), possibly executed via selective VLDL transcytosis by these cells. HDL lipoproteins exchange cholesterol and fatty acids with target cells. HDLs are rich in cholesteryl-esters, cholesterol is an important precursor for steroid production in the ovary, but also contain a relatively small amount of TAG. The total fatty acid concentration in follicular fluid is approximately 1.8-2.0 mM and is mostly present in lipoproteins esterified to cholesterol (cholesteryl-ester; 0.7 mM) and in fatty acids of the phospholipid layer of lipoproteins (1.1 mM; Jaspard *et al*., 1997; Valckx *et al*., 2014). During normal physiological conditions, (i.e. no metabolic stress) around 10% of the fatty acids present in follicular fluid is complexed to albumin as FFA (0.23 mM). During periods of metabolic stress, like energy scarcity, obesity or diabetes-type-II, the release of fatty acids from body fat is increased and results in elevated levels of FFA in blood and follicular fluid (Leroy *et al*., 2005; Aardema *et al*., 2013). Aberrant metabolic conditions do not appear to affect the HDL levels in blood and follicular fluid, as demonstrated in women with an increased body-mass-index (BMI) and in dairy cows that suffer from a metabolic stress condition compared with a control condition (Valckx *et al*., 2012; Aardema *et al*., 2013). In contrast, FFA levels massively increase in both blood and follicular fluid during metabolic stress conditions (Leroy *et al*., 2005; Aardema *et al*., 2013; Valckx *et al*., 2014). Whether the fatty acid composition of lipoprotein particles that reside in the follicular fluid is stable or changes during periods of metabolic stress is, to our knowledge, so far not known. It is also not clear whether the cumulus-oocyte-complex (COC) actively incorporates fatty acids that are present in lipoproteins. In this respect, the presence of CD36, a fatty acid translocase, on the cell membrane of cumulus cells and on the oocyte suggests that these cells are able to obtain fatty acids directly from follicular fluid (Uzbekova *et al*., 2015). The type of fatty acids taken up via CD36 depends on the length of the fatty acid; for instance, CD36 facilitates the uptake of long chain fatty acids when present in lipoproteins and of FFA. In both human and cow, the amount and molecular composition of FFA in follicular fluid varies dynamically according to the metabolic status of the individual (Aardema *et al*., 2013; Valckx *et al*., 2014). Although FFA forms only a small proportion of the total fatty acid pool in the follicular fluid, it is the most variable and hence metabolism dependent pool. Furthermore, the CD36 driven uptake of fatty acids by the COC is believed to be predominantly from FFA complexed to albumin (Hughes *et al*., 2011). Therefore, in this review we will focus on the impact of FFA on the oocyte.

## Neutral lipids are the major energy storage pool

Fatty acids can serve as an efficient energy source for cells. The complete aerobic catabolism of 1 mole of stearic acid versus 3 moles of glucose (for comparing in both cases 18 C atoms) yields 5-6 x more energy in the form of ATP. Fatty acids also form the main building blocks of cell membranes (phospholipids; the phosphatidyl-backbone of these lipids contains two fatty acids esterified to the sn-1 and sn-2 C atoms of glycerol) and lipid breakdown products can function as cell signalling molecules. In the oocyte, fatty acids can be packed as neutral lipid in the form of TAG (3 fatty acids esterified to glycerol) in lipid droplets. TAG, followed by phospholipids, form the most abundant class of lipid stored in the oocyte, furthermore, in contrast to most other cell types, TAG stored in the oocyte mostly contains saturated fatty acids (Homa *et al*., 1986; McEvoy *et al*., 2000; Kim *et al*., 2001; Aardema *et al*., 2013). The fatty acid composition of oocytes from Israeli Holstein cows shows seasonal variations, with an increased amount of unsaturated fatty acids during the winter period that causes a ∆T of 6°C in the melting temperature of membranes between summer and winter (Zeron *et al*., 2001). An increased level of unsaturated fatty acids lowers the melting temperature of membranes, phenomenon in cells to compensate for a reduced temperature, and improves membrane plasticity (Schumann, 2016). The fatty acid composition of cell membranes can be affected by fatty acids in the diet and during wintertime cows are mostly fed indoors on a ration of grass silage, corn and concentrates. During summer time, there is often supplemental outside grazing next to the ration indoors. Since grass feeding increases the uptake of unsaturated fatty acids, this option does not explain the higher level of unsaturated fatty acids in cell membranes during winter. Therefore, the effect of the dietary fatty acid change and effects on the oocyte during winter remains a yet unexplored but interesting area for future studies.

## Cumulus cells regulate the transfer of fatty acids to oocytes

**Figure 2:**
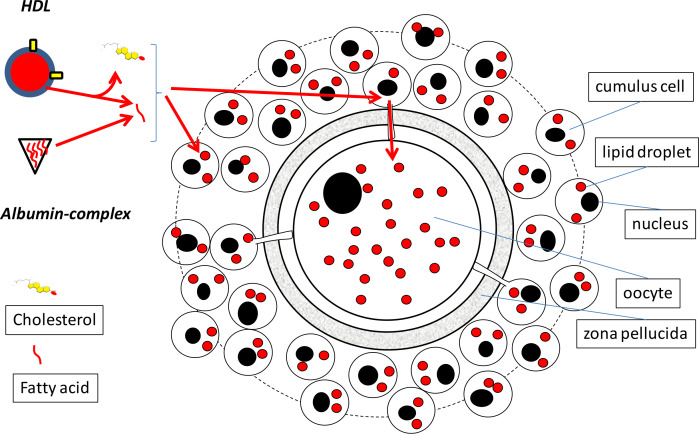
Delivery of fatty acids via HDL and albumin complex at the cumulus cell surface. HDL and Albumin (short hand depiction for details see Fig.1) can deliver their fatty acid via surface exchange to cumulus cells. Exchange of long chain fatty acids is facilitated by the presence of CD36 on the surface of cumulus cells. Cumulus cells can store the fatty acids in lipid droplets from where they can be metabolized for energy production or for building new membranes, or fatty acid may be transported by means of gap-junctional contact via protrusions of cumulus cells through the zona pellucida to the oocyte. The oocyte in turn can use these lipids for storage in lipid droplets or use them for energy by or for building new membranes. Note that besides fatty acids, HDL also exchanges cholesterol, which can be used for steroid production (cholesterol is a precursor). Red circles represent lipid droplets (for more details of them see Fig. 3).

Cumulus cells surrounding the oocyte form a natural barrier between follicular fluid and the oocyte. Before fatty acids can reach the oocyte, passage via cumulus cells appears to be unavoidable (Fig. 2). The question is whether and how the transfer of FFA from follicular fluid towards the cytoplasm of the oocyte takes place? It has been indicated that fatty acids are actively transferred from cumulus cells, which do express CD36 for the uptake of long chain fatty acids, to the oocyte (Uzbekova *et al*., 2015; Del Collado *et al*., 2017). The gap junctions, that via the zona pellucida connect cumulus cells and the oocyte and that are able to transfer small metabolites, do also contain fatty acid binding proteins (Dumesic *et al*., 2015; Russell *et al*., 2016; Del Collado *et al*., 2017). Interestingly, blockage of these transzonal projections with cytochalasin B resulted in a reduction of the lipid amount in the oocyte (Del Collado *et al*., 2017). A possible explanation by the authors was that oocytes showed decreased lipid amounts due to the reduction of fatty acid transport via the transzonal projections. This finding also shows that it is likely that diffusion of HDL of albumin FFA complexes through the intercellular matrix between cumulus cells of intact COCs is not sufficient for feeding the oocytes with fatty acids. Unfortunately, the fatty acid breakdown machinery in cytochalasin B treated oocytes was not studied. This could have excluded the possibility that the reduced lipid levels in the oocyte are due to increased fatty acid breakdown in the presence of cytochalasin B. Future studies are required to confirm the exclusive role of transzonal projections and their involvement in fatty acid transfer from cumulus cells to the oocyte.

Besides the facilitation of FFA transfer, cumulus cells are able to delay and hence to reduce the transfer of FFA towards the oocyte. Expanded cumulus cells of *in vivo* maturing COCs form a molecular filter that only allows restricted transfer of specific metabolites in the direction of the oocyte (Dunning *et al*., 2012). This proposed filter function of the cumulus layer supports the hypothesis that albumin complexed FFA and HDL -diffusion through the cumulus cell intercellular matrix of intact COCs, is not an important route for oocyte uptake of FFA (for the proposed major route for FFA entry in the oocyte see Fig. 2). The absence of a functional cumulus cell layer appears to simplify the entrance of exogenous fatty acids into the oocyte, as was demonstrated by an increased incorporation of exogenously administered fatty acids during *in vitro* maturation, into TAG in lipid droplets of oocytes that lacked an intact compact cumulus cell layer investment (Lolicato *et al*., 2015). Furthermore, in the presence of saturated stearic acid active removal of cumulus cells, after a first essential oocyte maturation period of 8 h with cumulus cells, resulted in a significant drop in the capacity of maturing oocytes to develop into a blastocyst when compared to intact COCs (Aardema *et al*., 2017). Cumulus cells massively accumulated fatty acids in lipid droplets after being exposed *in vivo* to elevated FFA levels in the follicular fluid of peri-ovulatory follicles, while no effect was measured on the lipid content nor on the developmental competence of the oocyte (Aardema *et al*., 2013). In somatic cell types lipid storage has been proven to be a protection mechanism to avoid cellular stress by fatty acids (Listenberger *et al*., 2003; Coll *et al*., 2008; Henique *et al*., 2010). This indicates that a proper functional expanded cumulus cell layer reduces the transfer of fatty acids and appears to protect the oocyte against abundant exposure to FFA. One may conclude that cumulus cells form an efficient barrier between FFA present in the follicular fluid and the oocyte. Whether or not cumulus cells are able to protect the developing oocyte against elevated levels of FFA during a prolonged period of time is not known and remains an interesting question, especially since metabolic aberrant conditions are most commonly experienced during an extended period of time.

## Lipid droplet dynamics in the oocyte

**Figure 3:**
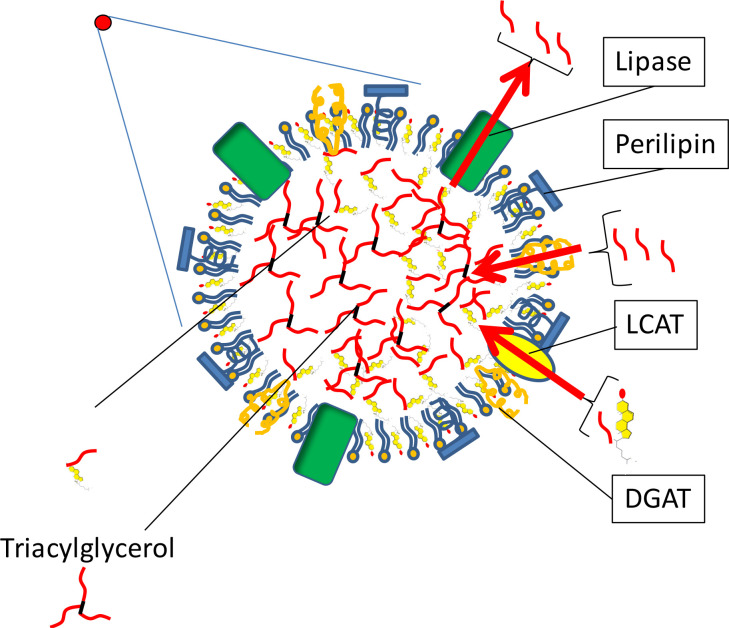
A lipid droplet is a dynamic organelle. Red circles (the lipid droplets from Fig. 2) are in this figure depicted with molecular details. Cholesterol and one fatty acid can be esterified to cholesteryl-ester by lecithin- cholesterol acyltransferase (LCAT) and fatty acids can be esterified to triacylglycerol (last step at the surface of the lipid droplet by the enzyme diacylglycerol acyltransferase (DGAT). Fatty acids from neutral lipids can be hydrolyzed and transported into the cytosol by the hormone sensitive lipase.

Physiologically, a high amount of lipid droplets is present in porcine, equine and bovine oocytes (Genicot *et al*., 2005; Sturmey *et al*., 2006; Ambruosi *et al*., 2009; Aardema *et al*., 2011). The abundant amount of neutral lipids stored in those lipid droplets seems to serve as a reserve energy deposit for developing oocytes and hence early-stage embryos. This was indicated by induced embryo development *in vitro* when fatty acid breakdown was stimulated and another study that demonstrated development of embryos in the complete absence of nutrients (Kane, 1987; Dunning *et al*., 2010; Sutton-McDowall *et al*., 2012). There appears to be a large variation in the amount of lipid droplets both in oocytes from different mammalian species and even within individual animals (Sturmey *et al*., 2009). A low number of lipid droplets is present in oocytes from mice, and an increasing number is noted for respectively human, sheep, bovine, horse and in porcine oocytes a very high number is present (Genicot *et al*., 2005; Aardema *et al*., 2011; Dunning *et al*., 2014). The differences that exist between mammalian species are thought to correlate with the elaborated duration and thus energy need of the embryo until functional nourishment from the placenta is taken over the energy demands of the implanted embryo (Sturmey *et al*., 2009). This period of time is short in rodents and primates and long in ruminants, equids and pigs; as a consequence, oocytes from the latter mammalian species contain high amounts of stored neutral lipids. An intriguing suggestion, because it suggests a controlled regulation of lipid storage in the oocyte that could be affected by exogenous exposure to certain fatty acids, or by altering lipid metabolism in the oocyte. Lipid droplets are formed at the endoplasmic reticulum and contain a hydrophobic core of neutral lipid, from TAG and cholesterol-esters, surrounded by a monolayer of phospholipids to meet the hydrophobic content with the hydrophilic nature of the ooplasm (Brown, 2001; Robenek *et al*., 2006). Enzymes of the perilipin family are located at the surface of the lipid droplet to control the storage and release of fatty acids from the lipid droplets (Fig. 3). The surface of lipid droplets in bovine and murine oocytes contains perilipin-2 (formerly named ADRP), a ubiquitously present perilipin; in addition to perilipin-2, perilipin-3 (formerly named TIP47), has also been localized to lipid droplets of porcine oocytes (Aardema *et al*., 2011; Yang *et al*., 2012; Uzbekova *et al*., 2015; Xu *et al*., 2018). Forty- five years ago, lipid droplets were, for the first time, recognized as potential metabolic units, due to their localisation next to mitochondria in the oocyte (Fleming and Saacke, 1972; Kruip *et al*., 1983; Hyttel, *et al*., 1997). The intimate relationship between lipid droplets and mitochondria has been confirmed by fluorescence resonance energy transfer (imaging, which indicates co- localization on a molecular scale of 6-10 nm (Sturmey *et al*., 2006). Fatty acid breakdown, β-oxidation, in mitochondria appears to be of fundamental importance for proper oocyte development (Downs *et al*., 2009; Dunning *et al*., 2010). Inhibition of fatty acid breakdown in the cumulus-oocyte-complex (COC) during IVM, by blocking the entry of fatty acids in mitochondria via the carnitine shuttle with methyl- palmoxirate or etomoxir, results in a sharp decline in the competence of murine, porcine and bovine oocytes to develop into an embryo (Ferguson and Leese 2006; Sturmey *et al*., 2006; Downs *et al*., 2009; Dunning *et al*., 2010;Paczkowski *et al*., 2013). However, studies where fatty acid breakdown in COCs was inhibited, did not discriminate between whether or not the inhibition was specific for cumulus cells and/or for the oocyte. The activity of carnitine-palmitoyl-transferase-I (CPT-I) is rate limiting for the transport of fatty acids into the matrix of the mitochondria and thus also rate limiting for the follow up β-oxidation of the transported fatty acids. The CPT-I activity is significantly higher in cumulus cells when compared to oocytes. Interestingly, when β-oxidation in COCs is stimulated by supplementation of the co-factor L-carnitine an improved developmental competence of the oocyte was observed (Dunning *et al*., 2011, 2014; Somfai *et al*., 2011). The positive association between the level of β- oxidation in COCs and the developmental competence of the oocyte may result from an abundant energy yield in the cell. However, another option for this positive association may be due to the breaking down and hence reduction of potentially toxic fatty acids.

## Sensitivity of oocytes for their lipid environment

Obesity in women is an example of a severe metabolic stress condition associated with elevated, potentially toxic, levels of FFA in blood and follicular fluid. Mouse oocytes exposed to lipid rich follicular fluid of obese women during IVM showed an increased amount of intracellular lipid; likewise oocytes retrieved from obese mice demonstrated a higher amount of lipid compared to controls (Wu *et al*., 2010; Yang *et al*., 2012). This suggests increased lipid uptake by oocytes during exposure to elevated levels of FFA. However, in contrast, it was striking that elevated levels of FFA in bovine follicular fluid during a period of negative energy balance did not affect the lipid content of *in vivo* derived oocytes (Aardema *et al*., 2013). While the exposure of *in vitro* maturing bovine COCs to elevated levels of the dominating follicular fluid -FFA resulted in rapid incorporation of fatty acids in lipid droplets in the oocytes (Aardema *et al*., 2015; Lolicato *et al*., 2015). The response of the oocyte to exogenous FFA thus seems to be unpredictable. However, a major metabolic difference exists between the above-described metabolic conditions. During obesity in an energy rich anabolic condition, glucose levels are high in blood and in follicular fluid and likewise during *in vitro* maturation glucose levels in the media are standard high. This situation is in sharp contrast to the catabolic negative energy balance of high yielding dairy cows, when the plasma levels of glucose are low. These distinct glucose concentrations that emerge during a condition with elevated levels of FFA may result in a different response of the oocyte to elevated levels of fatty acids, since the level of glucose coupled to insulin is the main driver for the energy storage in cells. Furthermore, the expression of CD36, the enzyme involved in the extracellular uptake of fatty acids by somatic cells, which is also expressed in both cumulus cells and oocytes, increases in response to elevated glucose and insulin levels (Uzbekova *et al*., 2015; Garbacz *et al*., 2016; Wilson *et al*., 2016; Ly *et al*., 2017). Indeed, it has been suggested that CD36 functions as a protective metabolic sensor in the liver during conditions of lipid overload. Storage of exogenous fatty acids by the oocyte, in the presence of elevated FFA in follicular fluid, thus appears to depend on glucose and consequently on insulin levels. The subsequent impact of elevated levels of FFA on the oocyte may therefore largely depend on the energetic condition of the dam. Furthermore, there appears to be a significant difference in the response of oocytes to distinct classes of FFA: 1) Exposure of bovine COCs to blood and follicular fluid - dominating saturated FFA (palmitic and stearic acid) during *in vitro* maturation resulted in a significant reduction in the number of lipid droplets in the oocyte and had a negative impact on the developmental competence of the oocyte (Aardema *et al*., 2011). 2) In contrast, exposure to the dominating mono-unsaturated oleic acid caused increased numbers of lipid droplets in the oocyte and counteracted the adverse effects of saturated fatty acids by restoring the number of lipid droplets and the developmental competence of the oocyte (Aardema *et al*., 2011). Another study demonstrated that oocytes with a higher intracellular lipid level of oleic acid appear to have increased developmental competence in comparison to oocytes with a higher level of stearic acid (Kim *et al*., 2001). This indicates that apart from an either energy rich or poor metabolic condition, the type and balance of saturated and unsaturated FFA appears to be of fundamental importance to the impact of FFA on oocyte developmental competence.

## Saturated free fatty acids induce lipotoxic stress responses in the COC

Saturated FFA appear to have a detrimental impact on the cumulus cells of the COC resulting in increased levels of apoptosis and hence a reduced developmental competence of the oocyte (Aardema *et al*., 2011, 2017; Leroy *et al*., 2005; Mu *et al*., 2001). Likely, the loss of viable and functional cumulus cells due to apoptosis impairs the ‘natural barrier’ of cumulus cells, which increases the risk for access of undesired FFA to the oocyte.

Maturing mouse COCs that are exposed to saturated palmitic acid experience a dose-dependent endoplasmic reticulum (ER) stress response, demonstrated by a rise in ER stress markers Atf4, Atf6, Xbp1s and Hspa5 and a reduced mitochondrial membrane potential, indicative for mitochondrial damage (Mu *et al*., 2001). The link between exposure to saturated FFA and lipotoxic events in COCs has also been demonstrated in other somatic cell types (Mu *et al*., 2001; Listenberger *et al*., 2003; Coll *et al*., 2008; Henique *et al*., 2010). The induction of ER stress has been shown to be an important factor in the negative cascade of lipotoxic responses induced by saturated fatty acids. Moreover, ER stress markers are also increased in granulosa cells of obese women and in COCs exposed to a lipid rich follicular fluid of obese women (Wu *et al*., 2010; Yang *et al*., 2012; Sutton- McDowall *et al*., 2016). This observation suggests that saturated FFA induce lipotoxic responses of granulosa cells and the COC during obesity. Apart from inducing ER stress in COCs, saturated fatty acids also increase the level of the apoptosis inducer ceramide and ROS (reactive oxygen species; Lolicato *et al*., 2015). The increased level of ROS is a consequence of electron leakage from the inner mitochondrial membrane during oxidative phosphorylation (Dumesic *et al*., 2015).

Saturated palmitic acid is a potent inducer of an ER stress response and the resulting leakage of ER calcium stores triggers a cascade of intracellular actions, including unfolding and/or misfolding of proteins in the ER, that results in ROS formation in ER and mitochondria and consequently mitochondrial dysfunction (Pizzo and Pozzan, 2007; Ly *et al*., 2017). Due to mitochondrial damage and the consequently reduced fatty acid breakdown, the toxic impact of fatty acids for the cell is even further increased, which can eventually result in apoptotic cell death after the release of cytochrome-C. On the one hand β-oxidation has been associated with an undesirable increase of ROS production in mitochondria, however, on the other hand a reduction in lipotoxic responses has been observed when β-oxidation was stimulated (Henique *et al*., 2010; Ly *et al*., 2017).

**Figure 4:**
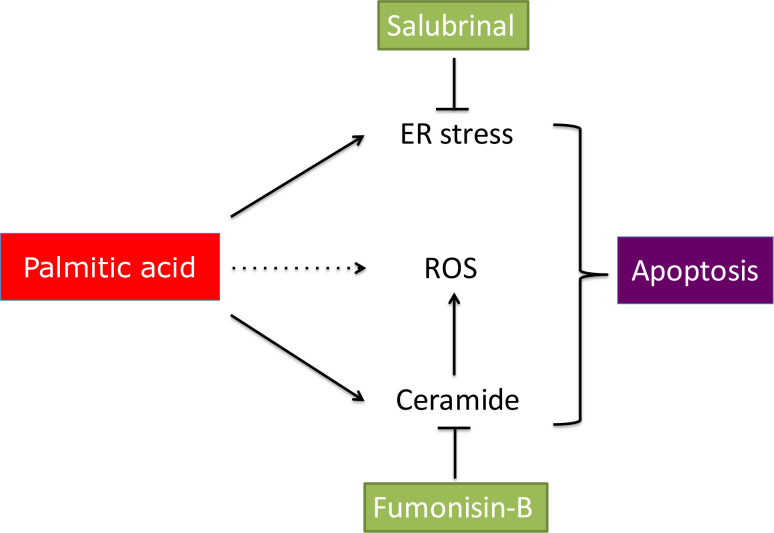
Saturated FFA induce lipotoxic events in COCs and reduce oocyte developmental competence. ER stress and mitochondrial damage are key events in the cascade of actions induced by saturated FFA in COCs. Lipotoxic events can be prevented by inhibition at two points (i) inhibition of ER stress by salubrinal and inhibition of ceramide formation by fumonisin-B.

The cascade of actions induced by saturated FFA can be prevented by inhibition at two points (i) inhibition of ER stress by salubrinal in the presence of palmitic acid during IVM results in improved mitochondrial function and cumulus cell morphology and prevents a negative impact on oocyte developmental competence (Wu *et al*., 2012). This indicates that the ER is one of the primary harmed organelles in COCs exposed to palmitic acid, in line with the observed responses towards palmitic acid exposure in other somatic cell types. (ii) Inhibition of ceramide formation by fumonisin-B results in a significant reduction in the ROS formation and a reduction in cumulus cell deterioration and increased cumulus cell expansion, which suggests that the pro- apoptotic ceramide is an important initiator of ROS generation (Lolicato *et al*., 2015). Both ER stress and ceramide formation appear to be key events in the lipotoxic response observed in COCs exposed to palmitic acid (Fig. 4).

The role of CD36 for COCs in relation to their response to palmitic acid may be of interest for future research. Inhibition of CD36 in somatic cell types reduces ROS formation and prevents lipotoxic events during exposure to palmitic acid (Hua *et al*., 2015; Xu *et al*., 2015; Kim *et al*., 2017). Whether or not CD36 is also involved in the cascade of lipotoxic events induced by saturated FFA in COCs needs to be elucidated. Another interesting option to prevent a potential toxic impact by saturated FFA is the safe storage of fatty acids in lipid droplets of the cell, which is stimulated by mono-unsaturated FFA (Listenberger *et al*., 2003; Coll *et al*., 2008; Henique *et al*., 2010).

## Mono-unsaturated oleic acid prevents saturated fatty acid stress on oocyte

Mono-unsaturated oleic acid is in contrast to the response of COCs to saturated fatty acids, harmless to COCs and the oocytes. During exposure to high concentrations of oleic acid, oocytes of exposed COCs remain fully competent to develop into an embryo (Leroy *et al*., 2005; Aardema *et al*., 2011; Wu *et al*., 2012). The distinct impact of mono-unsaturated fatty acid versus saturated on cells has been attributed to a different intracellular distribution of the fatty acids. Mono-unsaturated fatty acids like oleic and palmitoleic acid are primarily distributed towards lipid droplets, whereas, saturated fatty acids are directed to apoptotic pathways in somatic cell types (Listenberger *et al*., 2003; Coll *et al*., 2008; Henique *et al*., 2010). Oleic acid does not induce a significant ER stress response in somatic cells, unlike palmitic acid (Ly *et al*., 2017). Interestingly, when oocytes are simultaneously exposed to oleic acid and saturated FFA the negative impact of the saturated fatty acids alone on the oocyte is counteracted and oocyte developmental competence is maintained (Aardema *et al*., 2011). A recent paper demonstrated that alpha-linolenic acid also appears to have the potency to prevent a lipotoxic impact by FFA on the oocyte (Marei *et al*., 2017). However, it is good to note that in contrast to oleic acid, which is not toxic even at high concentrations (500 µm), alpha-linolenic acid has a detrimental impact on oocyte developmental competence above a concentration of 50 µm (Marei *et al*., 2009; Aardema *et al*., 2011). The protective mechanism by which mono-unsaturated FFA reduce the potential stress of saturated fatty acids, is thought to be via redistribution away from apoptotic cascades (ROS and ceramide formation), towards lipid droplets for storage (Listenberger *et al*., 2003; Coll *et al*., 2008; Henique *et al*., 2010). Interestingly, follicular fluid contains a relatively high amount of oleic acid in comparison to the levels in blood (Leroy *et al*., 2005; Aardema *et al*., 2013). Cumulus cells of COCs exposed to elevated levels of FFA during a (mimicked) period of metabolic stress, both *in vivo* and *in vitro*, massively incorporate fatty acids in lipid droplets resulting in a 6- fold increase of TAG storage (composed primarily by esterified oleic acid); moreover, the oocytes of the exposed COCs remain fully competent to develop into an embryo (Aardema *et al*., 2013). Storage of fatty acids in the form of TAG in cumulus cells appears to be an effective route to prevent a toxic impact of FFA in the COC, which is in line with observations in other somatic cell types.

The impact of an elevated concentration of FFA during metabolic stress in cows was investigated *in vitro* by supplementing maturation media with comparable concentrations of the three dominating FFA, saturated palmitic and stearic and mono- unsaturated oleic acid, in two distinct experimental setups. Fatty acids were either solved in ethanol and were stirred (Van Hoeck *et al*., 2011; Sutton-McDowall *et al*., 2016) or were complexed to albumin, which is comparable to the physiological presentation of FFA to the COC (Aardema *et al*., 2013). COCs exposed to a mixture of saturated and mono-unsaturated fatty acids solved in ethanol, demonstrated hampered oocyte developmental competence (Van Hoeck *et al*., 2011; Sutton-McDowall *et al*., 2016), whereas, in the study of Aardema *et al*. no such negative impact of a comparable FFA mixture was observed in line with the observations after *in vivo* exposure of COCs to elevated levels of FFA during metabolic stress (Aardema *et al*., 2013). The expected presentation of fatty acids in micelle formation after ethanol injection or via the physiological presentation in a complex with albumin may account for the distinct outcome between the studies and this certainly needs further investigation. The absence of a negative impact on the oocyte competence after exposure of COCs to a mixture from saturated palmitic and stearic acid and mono-unsaturated oleic acid (Aardema *et al*., 2011; Aardema *et al*., 2013) is in line with observations in other cell types exposed to a combination of saturated and mono-unsaturated FFA (Mu *et al*., 2001; Listenberger *et al*., 2003; Henique *et al*., 2010). The observed distinct impact of saturated and mono-unsaturated FFA on oocyte developmental competence clearly indicates that a balance between the different types of FFA, rather than the total amount of FFA, appears to determine the impact on the COCs.

## The follicle creates a protective oleic rich FFA environment for the oocyte

The beneficial environment for the COC thus appears to be follicular fluid rich in mono-unsaturated oleic acid and, therefore, it is intriguing that the follicular fluid contains a relatively high level of mono- unsaturated oleic acid and a relatively low level of stearic acid, compared to blood (Aardema *et al*., 2011, 2013, 2015). Previously, it has been suggested that the distinct FFA composition of follicular fluid originates from selective transfer of FFA over the blood/follicle membrane. There are indeed indications that CD36/FAT (fatty acid translocase), the transmembrane protein that facilitates diffusion of long chain fatty acid, may be selective in the uptake of long chain fatty acids. In this respect, CD36/FAT is expressed in all cell types of the follicle including the oocyte itself (Uzbekova *et al*., 2015). Over expression of chicken CD36/FAT in Chinese hamster ovary cells resulted in an increased uptake of linoleic and arachidonic acid and a reduced uptake of palmitic acid, but overexpression of CD36 had no effect on the uptake of stearic and oleic acid (Guo *et al*., 2013). To this end, selective transfer of fatty acids over the blood-follicle membrane does not seem to explain the relatively high oleic acid levels in the follicular fluid. The distinct FFA composition in follicular fluid could originate from either storage or metabolism by the cells that form the follicle; theca, granulosa and cumulus cells all contain the intracellular machinery for fatty acid uptake and metabolism (Uzbekova *et al*., 2015). Fatty acids that dominate in theca cells, the cells that form the outer layer of the blood-follicle membrane, do reflect the composition of FFA in blood and are dominated by mono-unsaturated oleic acid (31%) and saturated palmitic (21%) and stearic acid (12%) in sheep (Hughes *et al*., 2011). Granulosa cells, which cells form the inner layer of the blood-follicle membrane, do also reflect dietary supplementation of n-3 or n-6 poly-unsaturated fatty acids (Wonnacott *et al*., 2010). As mentioned before, when COCs are exposed to elevated levels of FFA during IVM cumulus cells respond with intracellular storage of these fatty acids (Wonnacott *et al*., 2010; Aardema *et al*., 2013). This indicates that intracellular storage of fatty acids may affect the composition of FFA in follicular fluid, due to uptake of, formerly free, fatty acids by cells. However, the intracellular lipid that was stored in COCs exposed to elevated levels of FFA was dominated by oleic acid storage (Aardema *et al*., 2013). To this end, this phenomenon cannot explain the relatively high level of oleic acid in follicular fluid, as storage of oleic acid would result in a net reduced level of oleic acid in follicular fluid. Both selective uptake of FFA nor storage and metabolism do seem to provide an explanation for the distinct FFA composition in follicular fluid. There is, however, an interesting third option: the desaturation of saturated fatty acids into mono-unsaturated FFA in follicular fluid. The key enzyme responsible for the conversion of saturated stearic acid into mono-unsaturated oleic acid is SCD, i.e. by the formation of a double carbon bond at the ∆9 position of saturated fatty acid. In the rat ovary SCD type 2 is indeed expressed (SCD type 1-4 are present in murine species) in theca, granulosa and cumulus cells (Moreau *et al*., 2006). Women as well as cows do express SCD types 1 and 5 and both types are also expressed in granulosa and cumulus cells (Feuerstein *et al*., 2007; Aardema *et al*., 2017). Likely, the relatively low stearic and high oleic acid levels in follicular fluid, when compared to blood, may originate from SCD activity in the follicle (Aardema *et al*., 2013, 2015). After entrance of FFA from blood via the blood-follicle barrier, the active conversion of stearic acid into oleic acid in both theca and granulosa cells may explain the relative high level of mono-unsaturated FFA in follicular fluid, at the cost of saturated FFA. To this end, the transfer of FFA from blood to the follicle and the potential desaturation of FFA by follicular cells certainly needs attention in future research, in particular as being a promising route to potentially detoxify the FFA to which the COC is exposed.

## Cumulus cells protect the oocyte against potential stress by free fatty acids

**Figure 5:**
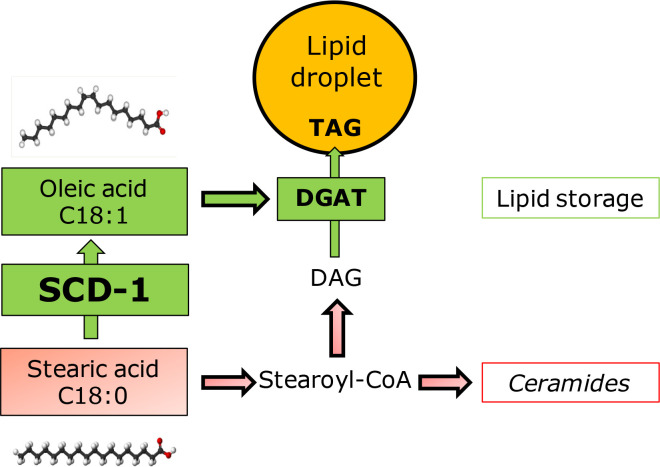
Cumulus cells protect the oocyte by SCD-1 activity and fatty acid storage. Saturated stearic acid induces apoptotic pathways. Conversion of saturated stearic into mono-unsaturated oleic acid by Stearoyl-CoA-Desaturase (SCD-1) and consequent storage of fatty acids in the lipid droplets of cumulus cells protects the oocyte against saturated fatty acids.

Interestingly, cumulus cells appear to be important in regulating the fatty acid transfer towards the oocyte and seem to protect the oocyte against potential stress by free fatty acids. In the absence of cumulus cells, oocytes appear to be extremely prone to elevated levels of FFA (Aardema *et al*., 2013; 2017;

Lolicato *et al*., 2015). We investigated whether the enzyme SCD-1, that can detoxify saturated FFA into mono-unsaturated FFA, is expressed in COCs and has a role in protecting the oocyte against FFA. The genes *SCD-1* and *SCD-5* are expressed in human cumulus cells and *SCD* expression was associated with oocyte competence (Feuerstein *et al*., 2007). *SCD-1* mRNA expression in human cumulus cells is increased in the presence of whole and partly delipidified serum, which may indicate that these conditions relate to the competence of the oocyte, as *SCD* expression in cumulus cells has been related with oocyte competence (Mardomi *et al*., 2018). The elevated *SCD* expression in cumulus cells may however also be a direct response to the medium and could reveal a potentially beneficial medium for the development of the oocyte, rather than being a direct marker for oocyte quality. More studies are needed to investigate how *SCD* expression in cumulus cells and oocyte competence are linked. *SCD-1* mRNA expression has also been demonstrated in bovine oocytes (Aardema *et al.*, 2018; Department of Farm Animal Health, Faculty of Veterinary Medicine, Utrecht University, The Netherlands; unpublished data) and cumulus cells (Aardema *et al*., 2017; Warzych *et al*., 2017a, b). Warzych *et al*., 2017a showed a correlation between FFA composition in follicular fluid and *SCD-1* gene expression in cumulus cells, which may also alter the fatty acids that are transported and metabolized in the oocyte. Bovine cumulus cells abundantly express SCD-1 protein, in contrast to the oocyte with non- detectable levels of SCD-1 protein (Aardema *et al*., 2017). Furthermore, when SCD activity in cumulus cells was inhibited during exposure to a physiological level of stearic acid, the developmental competence of the oocyte was hampered and comparable with the situation where oocytes were exposed to stearic acid in the absence of cumulus cells (Aardema *et al*., 2017). In a recent paper, the negative outcome of SCD inhibition in cumulus cells on oocyte developmental competence was also confirmed in human cumulus cells and also appeared to relate to reduced aromatase gene expression and estradiol production (Fayezi *et al*., 2018). SCD-1 expression has been associated with lipid metabolism in dairy cows and may be linked to reproductive performance (Wathes *et al*., 2012). SCD activity in cumulus cells appears to be of crucial importance to protect the oocyte against fatty acid stress. Furthermore, SCD activity in cumulus cells in the presence of stearic acid resulted in a significant reduction in the rate of apoptosis in the cumulus cells and hence increased lipid droplet storage of fatty acids dominated by oleic acid (Aardema *et al*., 2017). This convincingly demonstrates that the role of SCD activity and the resulting storage of fatty acids in lipid droplets of cumulus cells appears to be an important strategy to protect the oocyte against elevated FFA levels during metabolic stress (Fig. 5).

## Conclusion

The amount and molecular composition of FFA in blood correlate with those in follicular fluid of pre- ovulatory follicles and hence FFA levels increase in both fluids during periods of metabolic stress. The type of FFA to which the maturing COC is exposed largely dictates the impact of elevated FFA levels on the survival of the COC and competence of the oocyte. Mono-unsaturated FFA compensate for a negative impact of saturated FFA on the COC and maintain the developmental competence of the oocyte. Cumulus cells that surround the oocyte regulate fatty acid transfer towards the oocyte, and an intact cumulus cell layer appears to be essential to protect the oocyte effectively against undesired FFA. SCD activity of cumulus cells prevents FFA stress on the oocyte due to conversion of potentially toxic saturated FFA (stearic acid) into harmless mono-unsaturated FFA (oleic acid), which is safely stored in lipid droplets of cumulus cells. At this point it is unknown whether and how presumptive oocytes during stages of early (pre-antral) follicular development, which lack the presence of cumulus cells, are protected during metabolic stress and in the presence of elevated FFA levels. These observations are important directions for future research.

## References

[B1] Aardema H, Vos PL, Lolicato F, Roelen BA, Knijn HM, Vaandrager AB, Helms JB, Gadella BM. (2011). Oleic acid prevents detrimental effects of saturated fatty acids on bovine oocyte developmental competence. Biol Reprod.

[B2] Aardema H, Lolicato F, van de Lest CH, Brouwers JF, Vaandrager AB, van Tol HT, Roelen BA, Vos PL, Helms JB, Gadella BM. (2013). Bovine cumulus cells protect maturing oocytes from increased fatty acid levels by massive intracellular lipid storage. Biol Reprod.

[B3] Aardema H, Gadella BM, van de Lest CH, Brouwers JF, Stout TA, Roelen BA, Vos PL. (2015). Free fatty acid levels in fluid of dominant follicles at the preferred insemination time in dairy cows are not affected by early postpartum fatty acid stress. J Dairy Sci.

[B4] Aardema H, van Tol HTA, Wubbolts RW, Brouwers JFHM, Gadella BM, Roelen BAJ. (2017). Stearoyl-CoA desaturase activity in bovine cumulus cells protects the oocyte against saturated fatty acid stress. Biol Reprod.

[B5] Ambruosi B, Lacalandra GM, Iorga AI, De Santis T, Mugnier S, Matarrese R, Goudet G, Dell'aquila ME. (2009). Cytoplasmic lipid droplets and mitochondrial distribution in equine oocytes: Implications on oocyte maturation, fertilization and developmental competence after ICSI. Theriogenology.

[B6] Auclair S, Uzbekov R, Elis S, Sanchez L, Kireev I, Lardic L, Dalbies-Tran R, Uzbekova S. (2013). Absence of cumulus cells during in vitro maturation affects lipid metabolism in bovine oocytes. Am J Physiol Endocrinol Metab.

[B7] Broughton DE, Moley KH. (2017). Obesity and female infertility: potential mediators of obesity's impact. Fertil Steril.

[B8] Brown DA. (2001). Lipid droplets: proteins floating on a pool of fat. Curr Biol.

[B9] Cetica P, Pintos L, Dalvit G, Beconi M. (2002). Activity of key enzymes involved in glucose and triglyceride catabolism during bovine oocyte maturation in vitro. Reproduction.

[B10] Coll T, Eyre E, Rodriguez-Calvo R, Palomer X, Sanchez RM, Merlos M, Laguna JC, Vazquez- Carrera M. (2008). Oleate reverses palmitate-induced insulin resistance and inflammation in skeletal muscle cells. J Biol Chem.

[B11] Del Collado M, da Silveira JC, Sangalli JR, Andrade GM, Sousa LRDS, Silva LA, Meirelles FV, Perecin F. (2017). Fatty acid binding protein 3 and transzonal projections are involved in lipid accumulation during in vitro maturation of bovine oocytes. Sci Rep.

[B12] Downs SM, Mosey JL, Klinger J. (2009). Fatty acid oxidation and meiotic resumption in mouse oocytes. Mol Reprod Dev.

[B13] Dumesic DA, Meldrum DR, Katz-Jaffe MG, Krisher RL, Schoolcraft WB. (2015). Oocyte environment: follicular fluid and cumulus cells are critical for oocyte health. Fertil Steril.

[B14] Dunning KR, Cashman K, Russell DL, Thompson JG, Norman RJ, Robker RL. (2010). Beta-oxidation is essential for mouse oocyte developmental competence and early embryo development. Biol Reprod.

[B15] Dunning KR, Akison LK, Russell DL, Norman RJ, Robker RL. (2011). Increased beta-oxidation and improved oocyte developmental competence in response to l-carnitine during ovarian in vitro follicle development in mice. Biol Reprod.

[B16] Dunning KR, Watson LN, Sharkey DJ, Brown HM, Norman RJ, Thompson JG, Robker RL, Russell DL. (2012). Molecular filtration properties of the mouse expanded cumulus matrix: controlled supply of metabolites and extracellular signals to cumulus cells and the oocyte. Biol Reprod.

[B17] Dunning KR, Russell DL, Robker RL. (2014). Lipids and oocyte developmental competence: the role of fatty acids and beta-oxidation. Reproduction.

[B18] Fair T, Hulshof SC, Hyttel P, Greve T, Boland M. (1997). Oocyte ultrastructure in bovine primordial to early tertiary follicles. Anat Embryol (Berl).

[B19] Fayezi S, Ghaffari Novin M, Darabi M, Norouzian M, Nouri M, Farzadi L, Darabi M. (2018). Primary culture of human cumulus cells requires stearoyl- coenzyme a desaturase 1 activity for steroidogenesis and enhancing oocyte in vitro maturation. Reprod Sci.

[B20] Ferguson EM, Leese HJ. (2006). A potential role for triglyceride as an energy source during bovine oocyte maturation and early embryo development. Mol Reprod Dev.

[B21] Feuerstein P, Cadoret V, Dalbies-Tran R, Guerif F, Bidault R, Royere D. (2007). Gene expression in human cumulus cells: one approach to oocyte competence. Hum Reprod.

[B22] Fleming WN, Saacke RG. (1972). Fine structure of the bovine oocyte from the mature graafian follicle. J Reprod Fertil.

[B23] Fortune JE. (1994). Ovarian follicular growth and development in mammals. Biol Reprod.

[B24] Garbacz WG, Lu P, Miller TM, Poloyac SM, Eyre NS, Mayrhofer G, Xu M, Ren S, Xie W. (2016). Hepatic overexpression of CD36 improves glycogen homeostasis and attenuates high-fat diet-induced hepatic steatosis and insulin resistance. Mol Cell Biol.

[B25] Gautier T, Becker S, Drouineaud V, Menetrier F, Sagot P, Nofer JR, von Otte S, Lagrost L, Masson D, Tietge UJ. (2010). Human luteinized granulosa cells secrete apoB100-containing lipoproteins. J Lipid Res.

[B26] Genicot G, Leroy JL, Soom AV, Donnay I. (2005). The use of a fluorescent dye, Nile red, to evaluate the lipid content of single mammalian oocytes. Theriogenology.

[B27] Gosden RG, Hunter RH, Telfer E, Torrance C, Brown N. (1988). Physiological factors underlying the formation of ovarian follicular fluid. J Reprod Fertil.

[B28] Guo J, Shu G, Zhou L, Zhu X, Liao W, Wang S, Yang J, Zhou G, Xi Q, Gao P, Zhang Y, Zhang S, Yuan L, Jiang Q. (2013). Selective transport of long- chain fatty acids by FAT/CD36 in skeletal muscle of broilers. Animal.

[B29] Henique C, Mansouri A, Fumey G, Lenoir V, Girard J, Bouillaud F, Prip-Buus C, Cohen I. (2010). Increased mitochondrial fatty acid oxidation is sufficient to protect skeletal muscle cells from palmitate-induced apoptosis. J Biol Chem.

[B30] Homa ST, Racowsky C, McGaughey RW. (1986). Lipid analysis of immature pig oocytes. J Reprod Fertil.

[B31] Hua W, Huang HZ, Tan LT, Wan JM, Gui HB, Zhao L, Ruan XZ, Chen XM, Du XG. (2015). CD36 mediated fatty acid-induced podocyte apoptosis via oxidative stress. PLoS One.

[B32] Hughes J, Kwong WY, Li D, Salter AM, Lea RG, Sinclair KD. (2011). Effects of omega-3 and -6 polyunsaturated fatty acids on ovine follicular cell steroidogenesis, embryo development and molecular markers of fatty acid metabolism. Reproduction.

[B33] Hyttel P, Fair T, Callesen H, Greve T. (1997). Oocyte growth, capacitation and final maturation in cattle. Theriogenology.

[B34] Jaspard B, Fournier N, Vieitez G, Atger V, Barbaras R, Vieu C, Manent J, Chap H, Perret B, Collet X. (1997). Structural and functional comparison of HDL from homologous human plasma and follicular fluid. A model for extravascular fluid. Arterioscler Thromb Vasc Biol.

[B35] Jungheim ES, Macones GA, Odem RR, Patterson BW, Lanzendorf SE, Ratts VS, Moley KH. (2011). Associations between free fatty acids, cumulus oocyte complex morphology and ovarian function during in vitro fertilization. Fertil Steril.

[B36] Kane MT. (1987). Minimal nutrient requirements for culture of one-cell rabbit embryos. Biol Reprod.

[B37] Kim DH, Cho YM, Lee KH, Jeong SW, Kwon OJ. (2017). Oleate protects macrophages from palmitate- induced apoptosis through the downregulation of CD36 expression. Biochem Biophys Res Commun.

[B38] Kim JY, Kinoshita M, Ohnishi M, Fukui Y. (2001). Lipid and fatty acid analysis of fresh and frozen-thawed immature and in vitro matured bovine oocytes. Reproduction.

[B39] Kruip TAM, Cran DG, Beneden TH, Dieleman SJ. (1983). Structural changes in bovine oocytes during final maturation in vivo. Gamete Res.

[B40] Leroy JL, Vanholder T, Mateusen B, Christophe A, Opsomer G, de Kruif A, Genicot G, Van Soom A. (2005). Non-esterified fatty acids in follicular fluid of dairy cows and their effect on developmental capacity of bovine oocytes in vitro. Reproduction.

[B41] Listenberger LL, Han X, Lewis SE, Cases S, Farese RV, Ory DS, Schaffer JE (2003). Triglyceride accumulation protects against fatty acid-induced lipotoxicity. Proc Natl Acad Sci USA.

[B42] Lolicato F, Brouwers JF, de Lest CH, Wubbolts R, Aardema H, Priore P, Roelen BA, Helms JB, Gadella BM. (2015). The cumulus cell layer protects the bovine maturing oocyte against fatty acid-induced lipotoxicity. Biol Reprod.

[B43] Ly LD, Xu S, Choi SK, Ha CM, Thoudam T, Cha SK, Wiederkehr A, Wollheim CB, Lee IK, Park KS. (2017). Oxidative stress and calcium dysregulation by palmitate in type 2 diabetes. Exp Mol Med.

[B44] Mardomi A, Nouri M, Farzadi L, Zarghami N, Mehdizadeh A, Yousefi M, Shanebandi D, Shaaker M, Darabi M. (2018). Human charcoal-stripped serum supplementation enhances both the stearoyl-coenzyme a desaturase 1 activity of cumulus cells and the in vitro maturation of oocytes. Hum Fertil (Camb).

[B45] Marei WF, Wathes DC, Fouladi-Nashta AA. (2009). The effect of linolenic Acid on bovine oocyte maturation and development. Biol Reprod.

[B46] Marei WFA, De Bie J, Mohey-Elsaeed O, Wydooghe E, Bols PEJ, Leroy JLMR. (2017). Alpha-linolenic acid protects the developmental capacity of bovine cumulus- oocyte complexes matured under lipotoxic conditions in vitro. Biol Reprod.

[B47] McEvoy TG, Coull GD, Broadbent PJ, Hutchinson JS, Speake BK. (2000). Fatty acid composition of lipids in immature cattle, pig and sheep oocytes with intact zona pellucida. J Reprod Fertil.

[B48] McKeegan PJ, Sturmey RG. (2012). The role of fatty acids in oocyte and early embryo development. Reprod Fertil Dev.

[B49] Moreau C, Froment P, Tosca L, Moreau V, Dupont J. (2006). Expression and regulation of the SCD2 desaturase in the rat ovary. Biol Reprod.

[B50] Mu YM, Yanase T, Nishi Y, Tanaka A, Saito M, Jin CH, Mukasa C, Okabe T, Nomura M, Goto K, Nawata H. (2001). Saturated FFAs, palmitic acid and stearic acid, induce apoptosis in human granulosa cells. Endocrinology.

[B51] Paczkowski M, Silva E, Schoolcraft WB, Krisher RL. (2013). Comparative importance of Fatty Acid Beta- oxidation to nuclear maturation, gene expression, and glucose metabolism in mouse, bovine, and porcine cumulus oocyte complexes. Biol Reprod.

[B52] Pizzo P, Pozzan T. (2007). Mitochondria-endoplasmic reticulum choreography: structure and signaling dynamics. Trends Cell Biol.

[B53] Robenek H, Hofnagel O, Buers I, Robenek MJ, Troyer D, Severs NJ. (2006). Adipophilin-enriched domains in the ER membrane are sites of lipid droplet biogenesis. J Cell Sci.

[B54] Russell DL, Gilchrist RB, Brown HM, Thompson JG. (2016). Bidirectional communication between cumulus cells and the oocyte: Old hands and new players?. Theriogenology.

[B55] Schumann J. (2016). It is all about fluidity: Fatty acids and macrophage phagocytosis. Eur J Pharmacol.

[B56] Somfai T, Kaneda M, Akagi S, Watanabe S, Haraguchi S, Mizutani E, Dang-Nguyen TQ, Geshi M, Kikuchi K, Nagai T. (2011). Enhancement of lipid metabolism with L-carnitine during in vitro maturation improves nuclear maturation and cleavage ability of follicular porcine oocytes. Reprod Fertil Dev.

[B57] Sturmey RG, O'Toole PJ, Leese HJ. (2006). Fluorescence resonance energy transfer analysis of mitochondrial:lipid association in the porcine oocyte. Reproduction.

[B58] Sturmey RG, Reis A, Leese HJ, McEvoy TG. (2009). Role of fatty acids in energy provision during oocyte maturation and early embryo development. Reprod Domest Anim.

[B59] Sutton-McDowall ML, Feil D, Robker RL, Thompson JG, Dunning KR. (2012). Utilization of endogenous fatty acid stores for energy production in bovine preimplantation embryos. Theriogenology.

[B60] Sutton-McDowall ML, Wu LL, Purdey M, Abell AD, Goldys EM, MacMillan KL, Thompson JG, Robker RL. (2016). Nonesterified fatty acid-induced endoplasmic reticulum stress in cattle cumulus oocyte complexes alters cell metabolism and developmental competence. Biol Reprod.

[B61] Uzbekova S, Elis S, Teixeira-Gomes AP, Desmarchais A, Maillard V, Labas V. (2015). MALDI Mass spectrometry imaging of lipids and gene expression reveals differences in fatty acid metabolism between follicular compartments in porcine ovaries. Biology (Basel).

[B62] Vahratian A. (2009). Prevalence of overweight and obesity among women of childbearing age: results from the 2002 National Survey of Family Growth. Matern Child Health J.

[B63] Valckx SD, De Pauw I, De Neubourg D, Inion I, Berth M, Fransen E, Bols PE, Leroy JL. (2012). BMI- related metabolic composition of the follicular fluid of women undergoing assisted reproductive treatment and the consequences for oocyte and embryo quality. Hum Reprod.

[B64] Valckx SD, Arias-Alvarez M, De Pauw I, Fievez V, Vlaeminck B, Fransen E, Bols PE, Leroy JL. (2014). Fatty acid composition of the follicular fluid of normal weight, overweight and obese women undergoing assisted reproductive treatment: a descriptive cross- sectional study. Reprod Biol Endocrinol.

[B65] Van Hoeck V, Sturmey RG, Bermejo-Alvarez P, Rizos D, Gutierrez-Adan A, Leese HJ, Bols PE, Leroy JL. (2011). Elevated non-esterified fatty acid concentrations during bovine oocyte maturation compromise early embryo physiology. PLoS One.

[B66] Warzych E, Pawlak P, Pszczola M, Cieslak A, Lechniak D. (2017a). Prepubertal heifers versus cows:the differences in the follicular environment. Theriogenology.

[B67] Warzych E, Pawlak P, Pszczola M, Cieslak A, Madeja ZE, Lechniak D. (2017b). Interactions of bovine oocytes with follicular elements with respect to lipid metabolism. Anim Sci J.

[B68] Wathes DC, Clempson AM, Pollott GE. (2012). Associations between lipid metabolism and fertility in the dairy cow. Reprod Fertil Dev.

[B69] Wilson CG, Tran JL, Erion DM, Vera NB, Febbraio M, Weiss EJ. (2016). Hepatocyte-specific disruption of CD36 attenuates fatty liver and improves insulin sensitivity in HFD-fed mice. Endocrinology.

[B70] Wonnacott KE, Kwong WY, Hughes J, Salter AM, Lea RG, Garnsworthy PC, Sinclair KD. (2010). Dietary omega-3 and -6 polyunsaturated fatty acids affect the composition and development of sheep granulosa cells, oocytes and embryos. Reproduction.

[B71] Wu LL, Dunning KR, Yang X, Russell DL, Lane M, Norman RJ, Robker RL. (2010). High-fat diet causes lipotoxicity responses in cumulus-oocyte complexes and decreased fertilization rates. Endocrinology.

[B72] Wu LL, Russell DL, Norman RJ, Robker RL. (2012). Endoplasmic reticulum (ER) stress in cumulus-Oocyte complexes impairs pentraxin-3 secretion, mitochondrial membrane potential (∆Ψm), and embryo development. Mol Endocrinol.

[B73] Xu M, Zeng Y, Chi D, Si L, Qu X, Li J. (2018). The dynamic pattern of PLIN3 in pig oocytes and cumulus cells during in vitro maturation. Zygote.

[B74] Xu S, Nam SM, Kim JH, Das R, Choi SK, Nguyen TT, Quan X, Choi SJ, Chung CH, Lee EY, Lee IK, Wiederkehr A, Wollheim CB, Cha SK, Park KS. (2015). Palmitate induces ER calcium depletion and apoptosis in mouse podocytes subsequent to mitochondrial oxidative stress. Cell Death Dis.

[B75] Yang X, Wu LL, Chura LR, Liang X, Lane M, Norman RJ, Robker RL. (2012). Exposure to lipid-rich follicular fluid is associated with endoplasmic reticulum stress and impaired oocyte maturation in cumulus- oocyte complexes. Fertil Steril.

[B76] Zeron Y, Ocheretny A, Kedar O, Borochov A, Sklan D, Arav A. (2001). Seasonal changes in bovine fertility: relation to developmental competence of oocytes, membrane properties and fatty acid composition of follicles. Reproduction.

